# Factors associated with COVID-19 booster dose uptake in the Jazan region, Saudi Arabia: insights for policy improvement during pandemics

**DOI:** 10.3389/fpubh.2026.1863210

**Published:** 2026-08-13

**Authors:** Mohammed J. Almalki, Amani A. Alotaibi

**Affiliations:** 1 Department of Public Health, College of Nursing and Health Sciences, Jazan University, Jazan, Saudi Arabia; 2 Quality and Patient Safety Department, Jazan University Hospital, Jazan University, Jazan, Saudi Arabia

**Keywords:** booster dose, COVID-19, insights for policy, pandemics, Saudi Arabia, vaccine acceptance, vaccine hesitation, vaccine rejection

## Abstract

**Objectives:**

This study aimed to assess factors associated with COVID-19 booster dose uptake in the Jazan region, Saudi Arabia.

**Methods:**

A sample of 467 individuals participated in a cross-sectional online questionnaire between February and March 2022. Data were investigated using descriptive characteristics, Pearson's chi-square, and logistic regression tests.

**Results:**

Nearly 69.6% of respondents believed the booster dose to be necessary, while 16.3% disagreed, and 14.1% were unsure. Almost two-thirds (61.7%) of the respondents had received the booster dose, while 7.9% had booked a vaccination appointment; the remaining 30.4% had neither received nor scheduled any appointments. Furthermore, 57% of non-vaccinated respondents (n=102) planned to receive the booster dose, 19.0% did not plan to receive the vaccine (n=34), and the remaining 24.0% were hesitant (n=43). Receipt of the booster dose of the vaccine was associated with the respondents' attitude, gender, and having not been previously diagnosed with COVID-19 (*P* < 0.05). Men were significantly more than twice as likely to receive the booster dose compared to females, while individuals who had positive attitudes toward the booster dose were up to 5 times more likely to be vaccinated.

**Conclusions:**

The COVID-19 virus and similar viruses continue to be a possible health risk due to the potential emergence of new, severe variants in the future. Understanding vaccine hesitancy can help manage the acceptance of booster doses and guide the development of targeted awareness campaigns for future disease outbreaks.

## Introduction

The COVID-19 pandemic had a major impact on the world since its emergence in December 2019, including in Saudi Arabia. Around 841,469 cases and over 9,000 deaths have been reported in Saudi Arabia alone (1).

The Saudi government implemented several preventive measures in response to the pandemic, including lockdowns, the closure of schools and businesses, travel restrictions, limitations on religious gatherings, and a nationwide vaccination campaign (2–5). Nearly 73.0% of the Saudi population completed the primary course of COVID-19 vaccines; however, only 46.0% received a booster dose (1). The COVID-19 vaccine was crucial to reducing severe cases, hospitalizations, and deaths related to COVID-19 (6); however, its effectiveness declines 6 months after the second dose, making recipients susceptible to the virus once again (7). In addition, there have been several new variants of the COVID-19 virus, such as Omicron, Delta, and Alpha (8). Many more variants are expected to emerge, and managing the threat these novel strains pose will require continued efforts (9). In response to the continued evolution of the virus, booster doses have been introduced to control its spread and impact. A booster dose is a single additional dose of a COVID-19 vaccine that is administered following the primary doses to prolong immunity (10). Booster doses strengthen the immune system and protect the recipient against severe COVID-19 infections (11–13).

The Saudi government introduced booster doses in December 2021 in line with global efforts to combat COVID-19 (14). However, hesitancy toward the booster dose has remained a global challenge, and Saudi Arabia is no exception. Abouzid et al. (15) conducted a regional study to investigate COVID-19 booster vaccine acceptability in the Middle East and North Africa (MENA) and found that while 60.2% of respondents were open to receiving the booster dose, around 20.4% remained hesitant. Furthermore, Saudi individuals were more hesitant to receive the booster dose compared to citizens of other countries in the region. The main reasons for refusing a booster dose were safety concerns and the belief that booster doses were unnecessary (15). Another study on Saudi citizens found that only 22.6% of respondents had received the booster dose (16). Likewise, another cross-sectional survey conducted in Saudi Arabia revealed that the booster dose was viewed negatively by 36.2% of respondents (17). Almost half of respondents (49.8%) demonstrated hesitancy toward booster vaccinations, and this attitude was more prevalent among women and respondents aged 60 years or older.

These findings suggest that hesitancy toward COVID-19 booster vaccines continues to pose a significant challenge to public health. It is therefore crucial to investigate and address this issue, especially in under-researched communities. This study investigates factors associated with the COVID-19 booster dose uptake in the Jazan region, Saudi Arabia.

The Jazan region is located in southwest Saudi Arabia and is bordered by the Aseer region to the north and east, the Red Sea to the west, and Yemen to the south and southeast (18). The region has a total area of 40,457 km^2^ and is home to a population of about 1.4 million, of which a majority are Saudi citizens (*n* = 1,002,779; 71.4%) (18, 19). Jazan is currently experiencing significant growth across multiple sectors, including infrastructure, trade, economy, agriculture, tourism, and education, which has attracted more people to the region for work and settlement. According to the General Authority for Statistics, the population of the Jazan region increased by 32.2% between 2010 and 2022 (19).

## Materials and methods

### Population and sample size

Epi Info for Windows version 7.2.4 (20) was used to calculate an appropriate sample size based on a 95% confidence interval, a 5% margin of error, and a 50% response rate for a population of about one million residents (19). A sample of 384 was found to be sufficient. Nevertheless, all completed questionnaires were included to reduce any potential sampling biases. The criteria for participant eligibility included having Saudi citizenship, being 18 years or older, and being a resident of the Jazan region.

### Data collection

Data were collected between February and March 2022 through a cross-sectional online survey that took an average of 4 min to complete. The link to the survey was shared via communication apps and social media platforms; respondents were advised to further distribute it within their personal networks. The questionnaire was designed by the authors of this article and written in both English and Arabic. Two bilingual public health researchers reviewed both versions for similarity, quality, and face validity. The Arabic version was then presented to a test group to ensure clarity. Data were collected using the Arabic version, while the English version was used for data analysis and this publication.

This questionnaire consisted of three sections: demographic characteristics, questions on the respondent's perception of the COVID-19 booster dose, and an open-ended section. Demographic characteristics included age, gender, marital status, presence of dependents, education level, whether the respondent had previously been diagnosed with COVID-19, and place of residency. The section on the booster dose was composed of five questions. The respondents were asked to rate the importance of the booster dose as well as their intention to receive it. Responses were graded on a five-point Likert scale and ranged from “strongly disagree” to “strongly agree.” The third question investigated the vaccination status of the respondents using three options: “received,” “not received but booked,” and “not received and not booked.” The final two questions sought reasons as to why respondents had accepted or rejected the booster dose. Finally, the open-ended section allowed respondents to add any information that they felt would improve the interpretation of the results.

### Ethical approval

This study was conducted in accordance with the local legislation and institutional requirements. Ethical approval was granted by the Standing Committee for Scientific Research at Jazan University in Saudi Arabia (HAPO-10-Z-001, REC-43/06/111). The survey information page included details about the importance of the study, the voluntary nature of participation, data privacy measures, and instructions on how to complete the questionnaire. Respondents were required to provide electronic consent before starting the questionnaire. Non-consenting individuals were required to leave the survey page.

### Data analysis

The IBM SPSS Statistics software (Version 29) was used to manage and analyze the data. The descriptive statistics were presented for the respondents' characteristics and their attitudes and intentions toward the booster dose. A Pearson's chi-square test was used to investigate the association between the booster dose (receipt status and the intention to receive) as dependent variables and the demographic characteristics, including age, gender, marital status, presence of dependents, education level, whether the respondent was previously diagnosed with COVID-19, area of residency, and attitude toward the booster dose. Logistic regression tests were performed to assess the predictive quality of each independent variable. All of the independent variables were either dichotomous or recoded to be dichotomous. To improve the attitude and intention items rating scale structure, and for analytical purposes, the 5-point Likert responses were combined and recorded into a binary format. Responses of “agree” and “strongly agree” were classified as having a positive attitude or intention toward the booster dose, whereas all remaining responses including “neutral,” “disagree,” and “strongly disagree” were classified as having a negative attitude or intention. The dichotomized variable was used in data analyses, including logistic regression. A *p-*value less than 0.05 was assumed to be statistically significant.

## Results

### Demographic characteristics

The survey received 467 completed responses. A majority of respondents were male (70.2%) and aged between 18 and 30 years (62.5%). The mean age of the respondents was 29.47 (SD = 9.36, range 18–55). A full description of the demographic characteristics can be found in Table 1.

**Table 1:** Demographic characteristics of the respondents.

Variables	Categories	*N*	%
Age	18–30 years	292	62.5
	> 30 years	175	37.5
Gender	Female	139	29.8
	Male	328	70.2
Marital status	Unmarried	273	58.5
	Married	194	41.5
Presence of dependents	No	253	54.2
	Yes	214	45.8
Education degree	Diploma or less	195	41.8
	Bachelor's degree or higher	272	58.2
Diagnosed with COVID-19	No	280	60.0
	Yes	187	40.0
Place of residency	Rural	171	36.6
	Urban	296	63.4

### Acceptance of the COVID-19 booster dose

**Figure 1:**
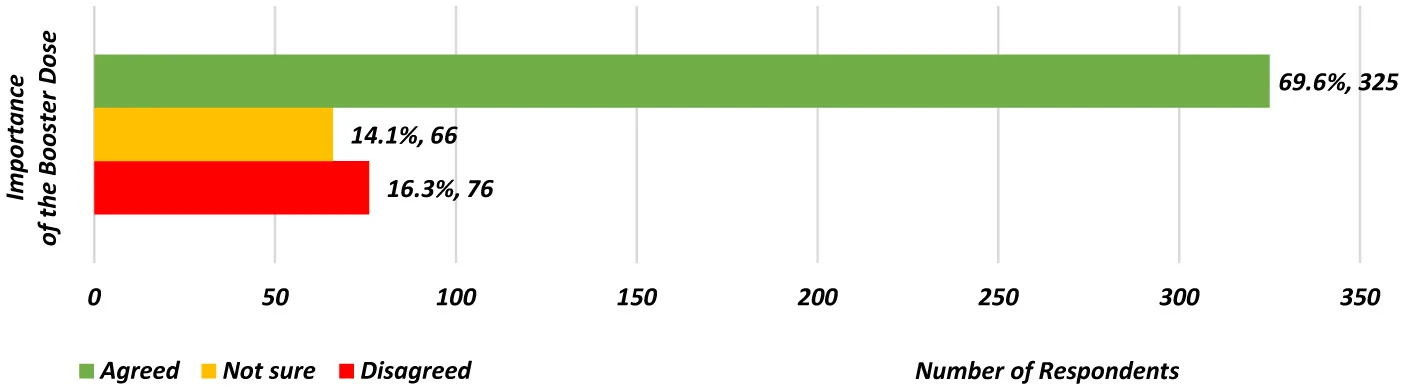
Respondents' attitudes toward the importance of the COVID-19 booster dose (*N* = 467).

**Figure 2:**
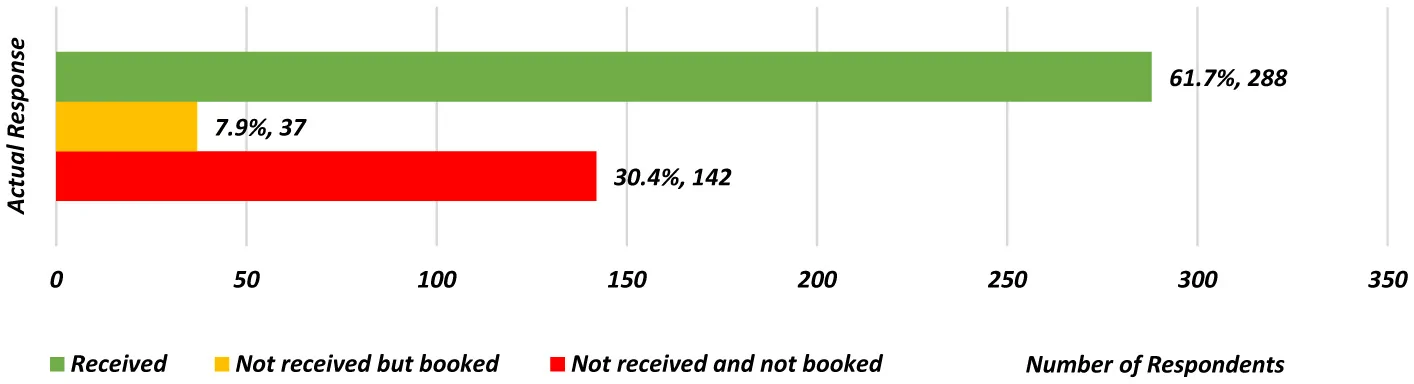
The vaccination status of the respondents with regards to the COVID-19 booster dose (*N* = 467).

When asked about the importance of receiving the booster dose, 69.6% of respondents agreed that it was important (*n* = 325), 16.3% disagreed (*n* = 76), and 14.1% were not sure (*n* = 66) (Figure 1). Nearly two-thirds of respondents (*n* = 288; 61.7%) had received the booster dose, while of those who had not received the booster dose, only 7.9% (*n* = 37) had booked a vaccination—the remaining 30.4% of respondents (*n* = 142) had not booked at all (Figure 2). When non-vaccinated respondents were asked if they intended to receive the booster dose, 57.0% agreed (*n* = 102), 19.0% disagreed (*n* = 34), and 24.0% were hesitant (*n* = 43).

**Figure 3:**
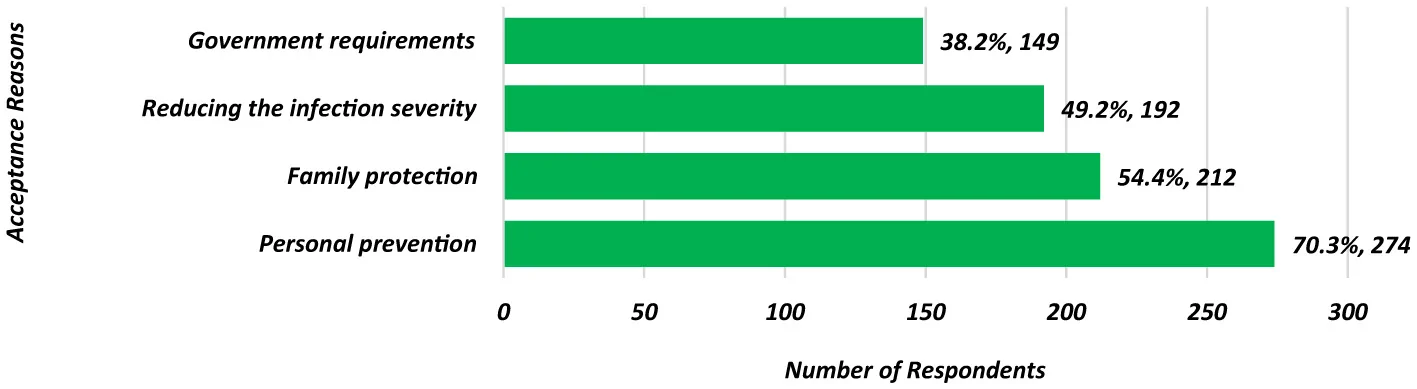
Reasons for accepting the COVID-19 booster dose (*n* = 390).

**Figure 4:**
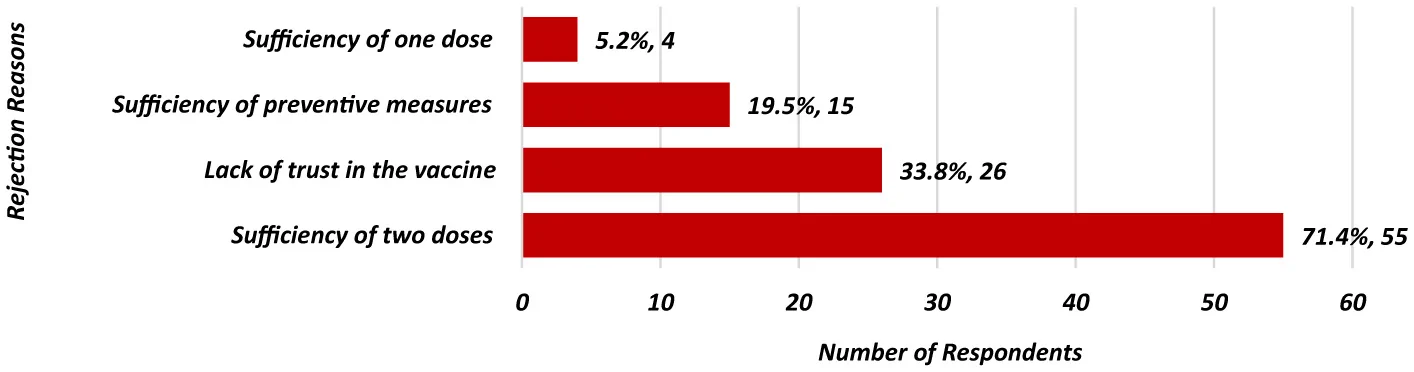
Reasons for the respondents' hesitancy or rejection of the COVID-19 booster dose (*n* = 77).

Figures 3, 4 present the reasons provided for the acceptance (*n* = 390) and hesitation/rejection (*n* = 77) of the booster dose among respondents. The primary reasons given for acceptance included personal prevention (*n* = 274; 70.3%), protecting families from COVID-19 (*n* = 212; 54.4%), controlling the severity of infection (*n* = 192; 49.2%), and complying with government requirements (*n* = 149; 38.2%). The primary reasons cited for the respondents' hesitancy or rejection (*n* = 77) included the perceived sufficiency of the two primary doses (*n* = 55; 71.4%), a lack of trust in the vaccine (*n* = 26; 33.8%), and sufficiency of preventive measures (*n* = 15; 19.5%).

### Relationship between receipt of the booster dose and the studied variables

Using Pearson's chi-square test, the respondent's attitude toward the booster dose showed the strongest association with the booster dose uptake (χ^2^= 70.47, df =1, *P* < 0.001) followed by gender (χ^2^= 16.85, df =1, *P* < 0.001), and whether the respondent had been previously diagnosed with COVID-19 (χ^2^=11.32, df =1, *P* < 0.001). Among the 288 respondents who indicated that they had received the booster dose, a majority (83.7%) agreed on the importance of the booster dose, 77.1% were male, and 66.0% had no prior history of COVID-19 (Table 2).

**Table 2:** Association between receipt of the COVID-19 booster dose and various demographic variables (*N* = 467).

Variables	Booster dose		Total		
	Not received (*n* = 179)	Received (*n* = 288)			
	*n* (%)	*n* (%)	*n*	χ^2a^	*P* value
Gender				16.85	<0.001^*^
Female	73 (40.8)	66 (22.9)	139		
Male	106 (59.2)	222 (77.1)	328		
Previously diagnosed with COVID-19				11.32	<0.001^*^
No	90 (50.3)	190 (66.0)	280		
Yes	89 (49.7)	98 (34.0)	187		
Attitude toward the importance of the booster dose				70.47	<0.001^*^
Disagree	95 (53.1)	47 (16.3)	142		
Agree	84 (46.9)	241 (83.7)	325		

*n* = number of respondents, % = percentage of respondents who had received the booster dose, a = Pearson's Chi-square Test, ^*^= statistically significant at *P* value <0.05.

Variables in which there was a significant association with receiving the booster dose at the univariate level *(P*<*0.05)* were further investigated using the binary logistic regression analysis. The respondents' gender, whether the respondent had been previously diagnosed with COVID-19, and the respondents' attitude toward the booster dose were assessed using this model. A Hosmer-Lemeshow goodness-of-fit test statistic revealed that the data were well-fit to the model *(P* = *0.73)*. A Nagelkerke R Square test showed that the predictor model accounted for 24.0% of the variability in recipients of the booster dose.

Significant associations *(P*<*0.05)* were found between the uptake of the booster dose and the respondent's attitude toward the booster dose (OR = 5.51, 95% CI: 3.55–8.55), gender (OR = 2.23, 95% CI: 1.43–3.48), and whether the respondent had been previously diagnosed with COVID-19 (OR = 0.58, 95% CI: 0.38–0.89). Respondents with a positive attitude toward the booster dose were more than five times more likely to seek a booster dose compared to those with a negative attitude. Men were more than twice as likely to receive a booster dose compared to females, and individuals previously diagnosed with COVID-19 were 1.7 times more likely to reject the booster dose compared to those without prior infection (Table 3).

**Table 3:** The results of binary logistic regression analysis on the selected independent variables associated with the COVID-19 booster dose uptake (*N* = 467).

Variable	B	Std. Error	Wald	df	Sig.	Odd ratio	95% CI	
							Lower	Upper
Gender	0.804	0.226	12.64	1	<0.001	2.230	1.434	3.478
Diagnosed with COVID-19	−0.539	0.213	6.372	1	0.012	0.583	0.384	0.887
Attitude toward the importance of the booster dose	1.701	0.224	57.997	1	<0.001	5.511	3.552	8.550

The dependent variable was the receipt of the booster dose. The independent variables were gender (female = 0, male = 1), whether the respondent had been previously diagnosed with COVID-19 (no = 0, yes = 1), and the respondents' attitude toward the importance of the booster dose (0 = disagree, 1 = agree). The reference category was presented first.

### Association between the intention to receive the booster dose and the studied variables

A Pearson's chi-square test revealed that only gender and attitude toward the booster dose were significantly associated (*P* < 0.05) with the intent to uptake the booster dose (*N* = 179). More than two-thirds (66.7%) of the respondents who reported the intention to receive the booster dose were males, and the vast majority (72.5%) indicated that they felt it was essential to receive the booster vaccine (Table 4).

**Table 4:** Association between the intention to receive the COVID-19 booster dose and the studied variables (*N* = 179).

Variables	Intention to receive the booster dose		Total		
	No (*n* = 77)	Yes (*n* = 102)			
	*n* (%)	*n* (%)	n	χ^2a^	*P* value
Gender				5.45	<0.015^*^
Female	39 (50.6)	34 (33.3)	73		
Male	38 (49.4)	68 (66.7)	106		
Attitude toward the importance of the booster dose				62.5	<0.001^*^
Disagree	67 (87.0)	28 (27.5)	95		
Agree	10 (13.0)	74 (72.5)	84		

*n* = number of respondents, % = percentage of respondents who had received the booster dose, a = Pearson's Chi-square Test, ^*^= statistically significant at *P* value <0.05.

A binary logistic regression model for the intention to receive the booster dose developed using the two aforementioned factors (gender and attitude) was found to produce satisfactory results (Table 5) and was capable of explaining 47.0% of the variation in the studied behavior. Males were three times more likely to intend to receive a booster dose compared to female respondents, while respondents with positive attitudes toward the booster dose were up to 21 times more likely to intend to receive the booster dose.

**Table 5:** The results of binary logistic regression analysis on the selected independent variables associated with the intention to receive the COVID-19 booster dose (*N* = 179).

Variable	B	Std. Error	Wald	df	Sig.	Odd ratio	95% CI	
							Lower	Upper
Gender	1.14	0.41	7.60	1	0.006	3.12	1.40	7.02
Attitude toward the importance of the booster dose	3.06	0.44	49.33	1	<0.001	21.24	9.05	49.18

The dependent variable was the intent to receive the booster dose. The independent variables were gender (female = 0, male = 1) and the respondent's attitude toward the importance of the booster dose (0 = disagree, 1 = agree). The reference category was presented first.

## Discussion

Saudi Arabia has taken several steps to combat COVID-19, including implementing preventive measures and policies, raising awareness of the virus, administering vaccinations, and providing adequate healthcare services. A majority of Saudi Arabians have received the primary doses of the COVID-19 vaccine (1). However, several people remain hesitant or refuse to receive the booster dose of the vaccine (16). The acceptance and rejection of COVID-19 vaccinations, including booster doses, vary widely across the globe (15, 21–24).

The current study found that two-thirds of respondents acknowledged the importance of receiving a booster shot against COVID-19 but only 61.7% of the respondents had received this dose. Among the unvaccinated respondents, only 57.0% reported that they were planning to receive the booster dose, while the remaining 43.0% were either hesitant or refused. The acceptance or rejection of booster vaccines is a complicated issue that is associated with individual attitudes and concerns.

The key reason cited by respondents for refusing the booster dose in the current study was the receipt of the two primary doses, which instilled a belief that these doses provided sufficient protection against COVID-19 and its complications. This result was consistent with previous studies in which individuals were less likely to take the booster after receiving the primary two doses (16, 25, 26). However, Alhasan et al. (27) reported contrary findings, with their results indicating that the receipt of the two primary doses was significantly associated with vaccine booster acceptance.

Another reason for refusing the booster dose was a lack of trust in the vaccine. Previous studies have found that many people lack confidence in the COVID-19 vaccine and the booster dose due to concerns about the vaccine's effectiveness, the possibility of contracting the disease after being vaccinated, and the potential side effects of vaccination (15, 16, 21, 28–30). A cross-sectional study conducted in Saudi Arabia revealed that around 95.0% of the respondents reported experiencing one or more side effects such as redness at the injection site, fatigue, body pains, fever, and headaches (31). Another study found that nearly two-thirds of their participants received medication to alleviate the severity of the side effects (32). These experiences tend to raise booster vaccine hesitancy rates (33).

A portion of the current study respondents also believed that preventive measures other than vaccination were sufficient to prevent COVID-19 infection; i.e., they believed that the vaccine, including its booster dose, was unnecessary. Despite the importance of preventive measures other than vaccination in combating COVID-19, several recent studies have emphasized the significant preventive role of vaccination, including the booster dose (11–13).

Respondents who were accepting of the booster doses reported several reasons for this perception. The primary reason was preventive purposes, both for personal and family protection. This finding was consistent with several studies conducted in Saudi Arabia, where respondents accepted the COVID-19 vaccine for prevention purposes (34, 35).

People in Saudi Arabia also took the COVID-19 vaccination to reduce the severity of potential infections and their related complications (28). This was an essential reason for the acceptance of the booster dose among many respondents of the current study. Health education, including awareness about the role that vaccination plays in mitigating complications and reducing the mortality rate of COVID-19, may influence people's decisions to accept booster doses. Several public awareness campaigns on COVID-19 and its vaccinations have been launched in Saudi Arabia (36, 37).

Many governments, including Saudi Arabia, implemented mandatory vaccinations for the general public to reduce the spread of COVID-19 (38). More than one-third of the current study respondents stated that this requirement was a primary reason for accepting the booster dose. Vellappally et al. (30) found that most of their respondents from India and Saudi Arabia had received the booster dose for similar reasons. Likewise, another study from Saudi Arabia revealed that 24.5% of respondents accepted the COVID-19 vaccine due to mandatory regulations (35).

This study found significant associations between the respondents' attitudes toward the booster dose and their receipt of or intention to receive the booster. Attitudes can have significant positive or negative effects on people's behavior (39). It is thus crucial to encourage positive personal attitudes toward COVID-19 preventive interventions, including booster shots. These interventions must also be considered during future pandemics. These findings are supported by recent evidence on attitudes toward the booster vaccine (40).

The respondents' gender was also associated with their receipt of or intention to receive the booster dose. Males were more likely to be accepting of the booster dose compared to females. However, the results of previous research on this topic have been inconsistent. Although several studies have reported that Saudi males were more likely to receive the booster dose compared to females (16, 28, 30), other studies found the opposite trend (17, 29, 41). Limbu and Huhmann (21) conducted a global systematic review and found that females were more likely to exhibit hesitancy toward booster doses compared to males. Global studies have indicated that gender differences in vaccine hesitancy are mediated by distinct factors across nations including culturally shaped health decision-making patterns, trust in healthcare systems, educational and employment status (42, 43). Future research should investigate the underlying demographic and sociocultural factors affecting gender differences in booster uptake within the Saudi community.

The results of this study also revealed that respondents diagnosed with COVID-19 were less likely to receive the booster dose. The primary reason for this hesitancy was feeling protected from the virus. A recent study from Saudi Arabia found that respondents who had not previously contracted the virus were more willing to accept the vaccine (35).

Previous studies have reported a higher acceptance rate of booster doses among urban residents compared to those in rural areas (15, 30, 41). However, the findings of the present study found no significant difference in booster dose acceptance rates based on residency. This could be due to the small size of the Jazan region (the second smallest region in Saudi Arabia); consequently, the entire region could be considered relatively urban. Another possible reason could be the nature of the sample, which consisted of educated individuals with Internet access.

## Insights for policy improvement during pandemics

It is important to examine the behavior of individuals who have refused or are hesitant to receive the booster vaccine as they may reject vaccinations during future pandemics. The risk of such behavior may influence the attitudes of other individuals toward vaccination. Consequently, promoting positive vaccine attitudes among the population is crucial to increasing vaccine uptake.

Efforts could be focused on women and those who have been infected, as these are the groups most likely to exhibit vaccine hesitancy. Women may have specific concerns, such as pregnancy or lactation (44) despite evidence showing that the vaccine, including booster shots, is safe and effective for pregnant and breastfeeding women (45). Individuals who had previously contracted COVID-19 were more likely to be hesitant toward or reject the vaccine due to a false sense of immunity (21). Some people may also experience side effects from the initial vaccine doses, leading them to avoid the booster shot. Previous research has reported on the many potential side effects of vaccinations (31, 32).

It is crucial to alleviate people's concerns regarding vaccines, including the booster dose, as well as address the relevant misinformation. This can be achieved by providing concise and updated information on the efficacy, safety, and importance of the vaccine and its booster doses. Health authorities can leverage recent technological advancements, including big data analytics and artificial intelligence, to address public health threats and enhance preventive measures. Artificial intelligence and big data provided insightful contributions during the COVID-19 pandemic (46–50). Saudi health authorities should utilize these tools to analyze the massive amount of available information and develop a robust system for raising public health awareness. This system would enable health authorities during outbreaks and pandemics to improve vaccination rates by targeting vulnerable individuals and hesitant demographics, customize education activities, and protect the nation's public health.

## Limitations and strengths

Due to the pandemic-related challenges, this study used a non-probability sampling technique via an online survey, which may introduced selection bias resulting in responses exclusively from males, younger and educated Saudi citizens with Internet access. In particular, this restricted the participation of females, older adults, and those with lower educational attainment or limited internet, which could influence the generalizability of the findings. However, both subsamples were large enough to conduct statistical tests. In addition, this study assessed a limited number of variables related to the behavior, while several other factors, such as socioeconomic status, occupation, trust in government and healthcare institutions, perceived risk of COVID-19 infection, and exposure to vaccine-related misinformation may play a significant role in the observed association. The lack of consideration of such variables may result in residual confounding factors, and this ought to be taken into account when interpreting the results.

Despite these limitations, the current study has contributed to the current knowledge of COVID-19 booster dose acceptance in Saudi Arabia, specifically in the Jazan region. The geographical location of Jazan, the occurrence of illegal border crossings, and its reputation as a port for pilgrims make the region vulnerable to health-related risks, especially during pandemics (51–54). The results of the present study are thus highly relevant to health authorities and those interested in the extent of booster vaccine acceptance in this region compared to the rest of Saudi Arabia and other countries. In addition, this information could help policymakers develop and prioritize appropriate preventive activities to encourage people to comply with preventive measures, including vaccine uptake, during pandemics.

## Conclusions

The present study found that about two-thirds of the respondents had reportedly received the booster dose, the remaining were either hesitant or refused. Attitude toward vaccination, gender, and prior COVID-19 infection history were significantly associated with receipt of the booster dose among participants. The strong association between positive attitudes and booster vaccination intention and uptake underlines the critical role of psychosocial factors in influencing vaccination behavior. The observed gender disparity, as men were significantly more than women to receive the booster dose, corroborates global evidence that documented the contextual variability in gender-related vaccine hesitancy. Future studies should explore factors shaping gender differences and the role of prior infection history in hesitancy toward booster vaccination to develop better tailored and effective vaccination strategies.

Despite the availability of free vaccinations and the health awareness campaigns launched by Saudi Arabia, some respondents were still hesitant to receive the booster dose. The most common reasons cited for this hesitancy by respondents included having already received the primary doses of the vaccination as well as a general distrust of the vaccine. As the COVID-19 virus (SARS-CoV-2) continues to evolve, the emergence of further pandemics may require vaccination as a preventive measure. It is therefore crucial to understand the reasons behind vaccine hesitancy among individuals to create targeted awareness campaigns as well as encourage the receipt of booster doses. Future research should consider underrepresented groups such as older people, expatriates, and those without Internet access to ensure they are accounted for.

## Data Availability

The raw data supporting the conclusions of this article will be made available by the authors, without undue reservation.
